# Letter from Dr. Harlan

**Published:** 1839-06-15

**Authors:** 


### FOREIGN CORRESPONDENCE.

### LETTER FROM DR. HARLAN.—No. III.

*Séances of the Royal Academy—M. Daguerre's Photogenic Discovery—Mortality among the Prostitutes of Paris—Epidemic among the Cows—Diseases of the Silk-Worms—French Surgeons—Mortality after Operations—M. Roux—Dr. Blandin's mode of treating Stumps after Amputation—Professor Breschet's Operation for Varicose Veins—Treatment of Fractures by the Immoveable Apparatus—M. Velpeau.*

Paris, April 30th, 1839.

To the Editors of the Medical Examiner.

Gentlemen,—Numerous pressing occupations, up to the present time, have effectually prevented the continuance of my correspondence. For the same reason, I have taken no notes of my daily observations, but hope, nevertheless, that some account of the general results may not prove altogether uninteresting to your readers. The weekly meetings of the National Institute—the daily exercises of the hospitals—the tri-weekly open days at the Jardin des Plantes, together with the urgent demands of Parisian society, leave me but little time for other occupations.

The Academy of Sciences, the only section of the Institute open to the public, holds it séances every Monday, at 3, P. M. The seats in the area outside those of the members, are always filled with visiters before half past 2, and those who arrive later are not admitted. This would have proved a great barrier to my attendance, had not the urbanity of my friend, Professor Blainville, of the Jardin des Plantes, secured me one of the eight chairs immediately before the President's chair, and devoted to foreign professors. The meetings are always well attended by the members; and more memoirs are usually presented than the Academy can receive, or the perpetual secretaries, M. Arago and M. Flourens, would be able to despatch. The crowds of savans, both foreign and native, who always claim admittance, shows the general interest that these séances excite.

Among the numerous interesting memoirs to the reading of which I have listened, none has so much riveted my attention as the account of the Photogenic discovery of M. *Daguerre*—the first annunciation of which was considered as fabulous. M. Daguerre's office, adjoining his splendid diorama near the Boulevard, was daily beset with the curious, demanding to know the truth of this new power of fixing an image,—the inventor was obliged, in self-defence, to close his doors,—this was just before my arrival in Paris, and previously to the reading of the memoir before the Academy of Sciences, where this highly important and interesting discovery occasioned much discussion and debate. I, however, enjoyed the rare opportunity of inspecting the portfolio of M. Daguerre, through the kind attentions of Mr. Walsh, by whom I was introduced. Whilst examining the unique productions of M. D.'s portfolio, and listening to his explanations, I felt as in the presence of a superior power. Among the principal productions of this new process presented to our admiration, I must mention, 1st, a view of the great gallery joining the Louvre to the Tuilleries; 2d, a view of l'Isle de la Cité,

| | |
|---|---|
| Hypertrophy of the heart, | 4 |
| Phthisis in the last stage, | 9 |
| Active Pneumonhagia, | 5 |
| Scirrhus of the stomach. | 3 |
| Hysteria in a high degree, | 4 |
| Hemiplegia, with cerebral congestion, | 3 |
| Chronic metritis, with fever, | 3 |
| Venereal Periostosis, | 3 |
| Rheumatism, with hectic fever, | 5 |
| Acute gout, | 2 |
| | 41 |

and the Towers of Notre Dame; 3d, views of the Seine, and several of its bridges; 4th, views of some of the Barrières of the Capital; 5th, views on the Boulevards; 6th, interior of the Chambers, with statuary, furniture, &c. These designs were of different epochs, from four years to four weeks, and were done at different seasons, and at various hours of the day, some by the light of the sun, some during a shower of rain, and some within doors, with a moderate light;—nothing could equal the beauty, accuracy, and perfection of these designs, which were equally magnificent when viewed by a magnifying glass, especially all *immoveable* objects. The process will not succeed with objects in constant motion; as an example, we observed a pair of carriage horses, in which one of the animals was headless, that part having been in continual motion. In answer to my question, what time was required for the entire process, M. D. stated that he could prepare his sensitive paper in two hours, and complete the design in from five to ten minutes,—or, continued he, "I only prepare the paper, and hold it up to nature, and she executes the drawing."

The time necessary for the execution of a view, when a great power of tone is expected, varies with the intensity of light, consequently the process is affected by the seasons, and even by the time of day, and by climate; in Egypt, for example, a view could be executed in one-third less time.

The process of M. Daguerre not only exacted the discovery of a substance more sensible to the action of light than any hitherto known to philosophers and chemists, it was also necessary to possess the means of depriving this substance of this new property at will, and M. D. has the merit of accomplishing this also. When his designs are once completed, they may be exposed to the direct rays of the sun, without undergoing any alteration. The extreme sensibility of M. D's. preparation is not the only character which distinguishes his discovery from those imperfect attempts formerly made to draw profiles on a layer of muriate of silver, which salt being white, is blackened by exposure to light, the white portions of the images becoming black, whilst the black portions, on the contrary, remain white. Upon the prepared screens of M. D., the drawing and the object are both similar—the white corresponding to white, the demi-tints to the demi-tints, the black to the black.

To demonstrate the extreme sensibility of M. D.'s preparation, he has succeeded in producing an evident white impression from the image of the moon, thrown through the focus of a moderate lens upon one of his prepared screens. A similar experiment was once made with the muriate of silver, without any effect, by MM. Laplace, Arago, and Malus.

The modus operandi in producing these designs, when the paper is once prepared, will be readily understood by all who are familiar with the camera obscura originally invented by Porta; every one has admired the neatness, truth of colour, form, and tone, with which exterior objects are reproduced upon the screen placed in the focus of the large lens which constitutes the essential part of this instrument, and have admired only to regret that these beautiful impressions could not be preserved. These regrets, together with the poetical expression—"fleeting as a shadow"—are henceforward without an object—M. D. having invented an artificial retina upon which the optical image leaves a perfect and lasting impression; or, in the language of M. Arago, "in the chambre noire of M. Daguerre, light itself reproduces the forms and proportions of exterior objects with a precision almost mathematical,—the photometrical relations of the various white, black, and gray parts, are exactly preserved,—but the red, yellow, green, &c., represent the demi-tints, for the new method creates designs, and not coloured pictures."

But, such designs! There is nothing in the arts that bears any analogous approach to them; the water is real water, the sky and clouds represent realities, the perspective and shading are the perfection of nature. The invention of M. Daguerre is the result of the assiduous application and labour of many years, during which he had as collaborator, M. Niépee, of Chalons-sur-Saône, recently dead. Some reclamations as to priority have been published by Mr. Talbot, of London, but he has failed in establishing his priority, and the results of his process are not similar to those of M. D.; in Mr. T.'s impressions the white portions of the image are black, and the black are white.

I regret to add that this inimitable portfolio of M. Daguerre, together with the splendid paintings constituting his diorama, was reduced to a mass of smoking ruins in three days after our visit; the premises took fire at 2 P. M., whilst M. Daguerre was on a visit to Professor Moss's, examining his Electro-Magnetic Telegraph, and as M. D.'s property was not insured, his prospects, at present, are ruinous.

My object in the present letter being to communicate results, rather than details, I must not enlarge further on other numerous and interesting memoirs presented to the Academy of Sciences; there is no subject, more or less related to science, that does not, some time or other, come under discussion at its séances. A curious fact was stated recently in a statistical memoir, viz.: only one case of disease out of thirty occurs among the prostitutes of Paris—one of the happy results of the sanitary laws, which exact a weekly surveillance of medical inspectors; but even this three and a half per centage is enough, in such a town as Paris, to fill two large hospitals,—and the disease in question is indubitably a prevalent one among the "gentlemen" of Paris. These, probably, obtain this proof of "attachment" from a class of society "above suspicion."

An "epizootic" or epidemical disease prevailed among the cows last winter in the neighbourhood of Paris; it was characterized by a contagious, vesicular eruption on the udder; it formed the basis of several memoirs of the professors of the Veterinary School at Alfort. The disease was never fatal, nor did it communicate any deleterious qualities to the milk, and it is only referred to now to show how much attention is paid to this interesting department of science by the savans of Paris. It is also recorded that the cows at Passy have recently been affected with the "cow-pock." The opportunity of fresh revaccination was improved with happy effect, the vaccine thus obtained being much more active. The question was started also, whether or not the cow was originally affected with this disease by inoculation from the human subject. I presume the experiment will sooner or later be made to inoculate the cow with the natural small-pox matter, and the modifying results noticed. We often listen to instructive memoirs on comparative anatomy, (especially fossil osteology,) geology, geography, rural economy, &c. &c., which, together with a very important memoir on the diseases of the silk worm, which destroys millions of worms in this country, and which consists in the growth or development of a species of cryptogamous vegetable of the genus Muscardine—the subcuticular adipose matter of the worm—we must refer to another opportunity.*

Parisian hospitals and French surgery might be presumed, *à priori*, to be the first objects of attraction to a practical surgeon; but I cannot but confess that a longer acquaintance with them, a

---

*Inasmuch as silk culture appears to have taken root at last in our country, and will soon, no doubt, add greatly to its agricultural resources, I may here remark, that excellent memoirs on the *Muscardine*, by Professor Audouin, have been communicated to the Academy of Sciences, and which were subsequently published in the "Annales des Sciences Naturelles" for October and November, 1837, January, 1838, and in the "Comptes Rendus" for 22d April, 1839. M. Bassi, of Lodi, was the first to announce, in 1835, the cause of this pestilence, so long the dread of the silk culturers of Italy and the South of France. When a colony of this useful insect is attacked, its ravages are instantaneous and general; none of those attacked have any chance of escape.

After numerous experiments, M. Audouin has arrived at the following conclusions, viz.:

"1st. That the Muscardine may be spontaneously developed, and in all places, when certain combined circumstances favour this development.

"2d. That it is not a disease confined to the silkworm, but that it is general, and may be exclusively peculiar to the class of insects.

"3d. That the disease, or Muscardine, may be propagated not only from silkworms to insects of very different species, but that, having spontaneously arisen among one of these species, it may, when transmitted to the silkworm, produce in them the same disease which shows itself in silk establishments, (magnaneries,) and which is designated under the name of '*Muscardine*.'

"4th. That during this transport, which may be indefinitely varied by operating upon insects of orders, families, genera, and species, different or alike, the cryptogamous plant, and the disease which it produces, experience no change.

"5th. That if the dissemination of the sporules in the air, be the means which nature employs for the reproduction of the plant, its artificial development may, nevertheless, be produced by inoculation of certain of its parts—its 'thallus,' for example—in the adipose tissue of the insect; that is to say, in the same soil in which the sporules have already vegetated.

"6th. In fine, by this artificial mode of infection, the cryptogame invades the adipose tissue much more rapidly, which occasions more promptly the death of the animal."

Subsequently, the following letter from a well-known agriculturalist, M. Poidebard, of the department of the Rhone, was communicated to the Academy of Sciences:—"There has resulted from the repeated experiments and observations which I have made in my magnanerie, (silk establishment,) a very important remark, viz.: a *magnanerie*, annually infested by the Muscardine, may, nevertheless, be made to yield a good harvest, by anticipating the period of the invasion and development of the disease by accelerating the education of the worms, which will allow them time to finish their cocoons before more extended course of investigation, and a more familiar intercourse with their most eminent teachers of surgery, have in no small degree lessened the admiration with which I once viewed the éclat generally attributed both to the men and the institutions. It is true, we cannot too much admire the long-continued and laborious application by which they have attained perfection as anatomists, and the consequent manual dexterity in operations, so universally admitted as a distinguishing characteristic of French surgeons,—and here their dexterity or superiority ends. Not only so: this dexterity itself has been obtained at the expense of principle, and at the expense of life; thousands are annually consigned to a premature grave by operations not always necessary to be performed at all, or improperly timed, or performed in cases that must terminate fatally, with or without operations. The mortality occurring at the Hôtel Dieu, perhaps one of the best, is absolutely frightful in amputations alone; the surgeons admit a loss of ninety-five per cent.; and one of the internes admitted, that during his residence for one year at the Hôtel Dieu, not one case of recovery occurred after amputation! I esteem M. Roux, the surgeon-in-chief of this extensive institution, as a personal acquaintance, and would not heedlessly detract from his hard-earned reputation; and in thus alluding to the results of my own personal observations, I have the interests of science only in view.

M. Roux possesses a grave, earnest, and decided character; but, like most others of his profession here, he is over-fond of displaying his manual dexterity. I have heard him beseech a patient to submit to an operation, as if it was the greatest favour conferred upon the operator; the operation once performed, the patient is pretty much consigned to his fate, for the after treatment of French surgeons I consider little better than no treatment at all. The constitutional demands, the habit, diathesis, or idiosyncrasies of the patient, are almost universally and entirely overlooked, and hence, together with the foul air of the hospitals, the dreadful mortality of these pest-houses. On the very first *coup d'œil* of the wards of these hospitals, nothing but disastrous consequences could be anticipated from one hundred to one hundred and fifty human sufferers, crowded together, side by side, and exhaling each the noxious effluvia peculiar to the gorgon form of diseases which afflict the inhabitants of rooms constructed on the worst possible principles for the purposes intended, and in which ventilation was not thought of, and where classification of disease has never been attempted. The unhappy effects of a foul atmosphere, in cases of wounds, are familiar to every professional reader,—as a remarkable instance of which, we may refer to the Dutch army of Batavia when attacked by the British navy, when every case of solution of continuity, however slight, resulted in gangrene. As a lecturer, M. Roux is animated, though by no means eloquent. During a private interview I held with him at his house the other day, he complained seriously, and lamented the state of French surgery of the present day in comparison with its former state; no one surgeon now, he said, could obtain half so many *operations* as formerly,—there were so many hospitals, and then each institution was "si partagé," "si isolé," there being eight or ten surgeons to each, and then almost every department of surgery being pursued in particular by some surgeon of eminence, that but few opportunities comparatively were left now days for the surgeon of a general hospital to show his skill. Only seven cases of lithotomy occurred in his own wards last year. Thus the diseases of the eye, the ear, hernia, club-foot, affections of the bladder and urinary organs, venereal disease, &c. &c., have each a hospital devoted exclusively to themselves.

---

the germ of contagion has made progress enough to occasion a general mortality.

"The following are the experiments on which my opinion is founded:

"1st. The silkworms of the white race, named *Sina*, whose term of existence does not exceed thirty days, reckoning from birth to the completion of the cocoon, have been constantly less injured than those of the *yellow* race, whose term of existence extends ten days longer.

"2d. The most precocious of the worms of the white race, were in no way affected by the disease.

"3d. Those a little tardy were affected, but in small numbers.

"4th. The more backward perished in much greater proportions.

"5th. Finally, the yellow silkworms, much more tardy, were nearly all destroyed."

It has been incontestably proved, that *contagion* is one of the most constant characters of the Muscardine.

I am convinced, Messrs. Editors, that you will not consider the above extracts foreign to the purposes of a medical journal; you know too well the extent to which the investigation of the diseases of the inferior animals has tended to the elucidation of the philosophy of life and disease in our own species.

M. R. is on the most familiar terms with his patients. In going his daily rounds from 7 to 9 A. M., he has always something funny, encouraging, or coaxing, to say to them all; for one he has a poke in the ribs with his finger, for another a box on the ear, &c. I have seen him make a convalescent reel with a blow on the side of his cheek, for asking him for something good to eat—all in the best possible humour. He sometimes becomes very affectionate, and kisses a patient on whom he has just inflicted a severe operation; and this salute, the students say, always prognosticates the death of the sufferer.

M. Roux mentioned to me Physick, Warren, and Mott, as the only American surgeons with whom he was acquainted; and, judging from them, said he wondered at the large and rapid fortunes that were accumulated by American surgeons—a consummation which he feared would never attend the efforts of Parisian surgeons.

In person, M. R. is rather beneath the ordinary stature, of a sanguino-phlegmatic temperament—features blunt and ill-favoured—one eye projects from its socket, and has a cast in it. When earnest in discourse, his countenance is especially contorted.

Dr. Blandin is also attached to the Hôtel Dieu. He has lately been coaxing his *stumps* to heal by the application of warm air after amputations, the stump being enclosed in a glass caisse, and air heated to the natural temperature of the body caused to pass constantly through it, is left without further dressing. Healing by adhesive inflammation, until within the last few years, was unknown in Parisian hospitals; from time to time some of the surgeons have attempted the adhesive process, but they are by no means fully aware of its importance. These stumps appeared to be granulating well, which may probably be accounted for by the simple circulation of air thus artificially produced. He has not yet published his results. It appeared to me rather an empirical practice.

Previously to leaving the Hôtel Dieu for the present, I must allude to Professor Breschet's method of operating for the radical cure of varicose veins. Your professional readers are already familiar with the construction of his instrument, consisting, briefly, in two parallel pieces of steel, brought together by screws, so as to press upon the included veins; the pressure is continued for several days, the patient keeping his bed; the integuments included above and below the veins slough; the veins are involved in the inflammation, and their sides adhere, obliterating their calibres. I have seen but one case involving the cord on both sides, in which the cure was apparently completed, the instrument being used on one side at a time.

On contrasting this operation with the method practised by M. Velpeau, of La Charité, I give the preference to the latter; it is less tedious, less complicated, less dangerous, and, what is no inconsiderable advantage, it is applicable to enlarged veins in any part of the body. M. V.'s method consists, simply, in passing a strong needle beneath the vein, and applying a ligature in circles, and not the figure of eight ligature. In a few days numerous veins are thus obliterated.

I have not been so forcibly impressed with any surgical improvement, since my arrival in Paris, as that of the treatment of fractures on the plan of Suetin and Velpeau, by means of "*Dextrine*" bandages. In my opinion, nothing but prejudice, superannuated or personal, can prevent the universal application of this method by all who are acquainted with it. Some of the Parisian surgeons have thus neglected it. At La Charité all other plans have long since been banished, and Velpeau and his assistants deserve much credit for the very great dexterity with which they so successfully treat all cases of fracture. There is, at present, a patient under treatment for fracture of the tibia and fibula, who was permitted to walk the ward in five or six days after its application. The same occurred since our arrival here, in a case of fracture of the os femoris, where the patient walked in fifteen days after the application of this bandage. These precocious efforts in similar cases are not, however, advisable. Its application to the treatment of fractured thigh is more effectual than any of the other methods, besides being so much more agreeable to the patient, and so much more speedy in its results, and so little troublesome to the surgeon, one single application being generally sufficient, especially if, before applying the bandages, sufficient time is allowed to permit the subsidence of all tumefaction; for this purpose, the patient is permitted to rest in an easy position for three or four days, when, if the bandage does not become loosened by the subsequent atrophy of the limb, a single application will suffice for the cure,—but even in case of reiterated application, it is less troublesome than the usual method.

The following is Velpeau's method of application in fractured os femoris: the limb is in the first place enveloped in a common roller; a piece of bandage is then crossed over the ankle, for the purpose of extension, and over these is placed another roller, thoroughly impregnated with the prepared paste; compresses of coarse paper, similarly soaked, are applied over the fracture, and other prepared rollers applied over these,—coarse pasteboard compresses, softened by similar soaking, are also applied, and kept in position by other rollers. The extension is now made by tying the bandage from the ankle to the foot of the bedstead, (which are all of iron in these hospitals,) and counter-extension is made by a strap passed between the thighs, and made fast to the bedstead above the head. The bandages harden in a few hours, and the limb is incased in a solid coat of mail.

Experiments have been made with numerous adhesive materials, such as starch, glue, paste, gum, &c., as substitutes for the "Dextrine" bandages now used exclusively at La Charité, all the others having been proved less effectual; gum arabic bears the nearest approach to it, but this is too expensive. The "Dextrine" is made by boiling starch in dilute nitric acid, and when separated from the liquor and dried, the powder costs about thirty cents a pound. One great advantage which this powder possesses over starch is, that it may be made into a paste with *cold* water, the former requiring boiling water. A very excellent and cheap substitute for linen or muslin bandages has been found in coarse paper, cut into suitable length and breadth, and soaked in this paste previously to their application; there can be nothing more effectual than a Scultet's bandage thus applied,—a method of economizing by no means beneath the notice of Parisian hospital surgeons, especially since a recent ordonnance of government, which obliges them to substitute paper for linen compresses—the savings thus accruing to be devoted to the gilding of that magnificent temple, the Madeleine church! A more detailed account of this "Dextrine" may be found by referring to "Orfila's Chemistry."

M. Velpeau has been most unfortunate in the loss of patients in La Charité, in which hospital the mortality has been greater than in any other establishment. In addition to the usual causes which have gained for this hospital so mortifying a distinction, there has prevailed during the last winter and present spring an erysipelatous diathesis, which has desolated its wards,—so that a puncture of a nail, the simple operation of extirpating a small ganglion from the neck of a healthy subject, the operation for the radical cure of varicocele, and amputations of all kinds, fractures, &c., I have seen terminate fatally in numerous cases; and yet M. Velpeau continued his operations, and appeared astonished himself at their want of success, and is far from taking advantage of the best means of averting the evil; his constitutional treatment is worse than nothing. A patient was admitted with a wound in the heel by a nail; erysipelatous inflammation followed, and was continued to the ankle-joint, producing suppuration, irritation, and death. No measures were resorted to in order to rally the powers of a broken constitution; and in the autopsy and lecture on this subject, M. V. expressed his inability to explain his want of success in the treatment of the case, and viewed the death of a patient from the simple puncture of a nail as an opprobrium to surgery! But human life, it is said, is of very little account with French surgeons. M. V. operated five times successively for disarticulation of the knee, and in every case his patients died.

But M. V. is a most dexterous and fearless operator; and if his hospital was more fortunately constituted, would doubtless be more successful. I have seen him twice remove tumours involving large portions of the lower jaw, with complete success; but he frequently operates needlessly and recklessly: he is more anxious to display his dexterity than to cure his patient. As a lecturer, M. Velpeau is quite too eloquent, the idea being lost in the midst of verbiage; hence, M. Lisfranc has given him the soubriquet of "le Paroquet," and sometimes he calls him "the Blacksmith"—M. V. having been educated to that trade—of which, however, he is in no manner ashamed, frequently alluding to the period of his life when he was "un maréchal." M. Velpeau's address is any thing but that of a gentleman. His personal appearance is vulgar, and his aspect forbidding; but his ugly countenance is agreeably relieved by a noble and expansive forehead, to which "oasis" he undoubtedly owes all his celebrity. He has a most disgusting habit of using the long, thin hair which covers his head, as a mop, on which to wipe his hands when soiled by some putrid piece of pathological anatomy on which he is expatiating before his class, every few minutes passing his fingers through his hair. It is to M. V.'s honour that he is never abusive of his confrères, though very frank in his intercourse with them, as is well exemplified in the following anecdote. Two of my friends, an English and American physician, called recently on M. V. at his office. On being ushered into an adjoining apartment, they overheard M. V. and another person altercating in the highest tone of voice; (this visiter proved to be Baron Heurteloup.) They could distinctly hear M. V. address the Baron in the following manner:—"Mais, M. le Baron, vous savez que vous êtes un grand menteur." When the Baron appeared to become furious, demanding to know what he meant by such an assertion, M. V. continued—"Soyez tranquille, soyez tranquille, M. le Baron; mais vous savez, entre nous, vous *savez* que vous êtes grand menteur!" Here the subject was dropped; M. le Baron, (who, by the way, I conceive to be too much of an adventurer,) not considering it any insult to be called by a name which he himself is very liberal in conferring on his confrères, especially as he was not aware that any third person was a witness to the scene.

